# Effects of Dexmedetomidine on the RhoA /ROCK/ Nox4 Signaling Pathway in Renal Fibrosis of Diabetic Rats

**DOI:** 10.1515/med-2019-0105

**Published:** 2019-11-20

**Authors:** Chen Jihua, Chen Cai, Bao Xubin, Yu Yue

**Affiliations:** 1 Department of anesthesiology, Fenghua District People’s Hospital, Ningbo City, Zhejiang Province, Philippines

**Keywords:** Dex, Diabetes, Renal fibrosis, RhoA/ROCK/Nox4, Rats

## Abstract

**Objective:**

To investigate the effects and mechanisms of dexmedetomidine (Dex) on model rats of diabetic nephropathy (DN).

**Methods:**

Rats were divided into NC, model, Dex-L (1μg/ kg), Dex-M (5μg/kg) and Dex-H (10μg/kg) groups. Rats in all groups except in the NC group were injected with streptozotocin (STZ) combined with right nephrectomy. Rats in Dex (1, 5 and 10μg/kg) groups received gavage with Dex (1, 5 and 10μg/kg). After 4 weeks, rats were sacrificed and kidneys were collected. HE staining was performed for a renal injury. Masson staining was applied to detect the fibrotic accumulation in rat kidney. Radioimmunoassay was used to test the renal function. Immunohistochemical method was used to detect protein expressions of RhoA, p-MYPT and Nox4 in rat kidney.

**Results:**

Compared with the NC group, the levels of urine microalbumin in protein, α1-MG and β2-MG, renal fibrotic accumulation, RhoA, p-MYPT, Nox4 and α-SMA in model group increased significantly (P＜0.001, respectively). Compared with the model group, Dex low, medium and high groups improved the deposition of renal fiber in rats, inhibited the expression levels of microalbumin, α1-MG and β2-MG in urine and decreased expression of RhoA, p-MYPT, Nox4 and α-SMA proteins (P＜0.05, P＜0.01).

**Conclusion:**

Dex is possible to inhibit the expression of α-SMA and renal fibrous substance deposition in rat kidney via RhoA/ROCK/Nox4 signaling pathway, thereby reducing early kidney damage in model rats.

## 1 Introduction

Diabetic nephropathy (DN) is one of the most common chronic complications of diabetes and is more common in developed countries [1]. It has been estimated that about a third of diabetic patients will eventually develop DN [1]. DN is characterized by proteinuria, glomerular hypertrophy, basement membrane thickening, podocytes reduction, protein deposition in the extracellular matrix, and most notably, by renal fibrosis [2, 3]. In recent years, the improvement in diabetes treatment has significantly reduced the mortality rate of patients due to acute complications of diabetes, but the incidence of chronic complications such as diabetic nephropathy has increased year by year [4]. Dexmedetomidine (Dex) is a highly selective α2 adrenergic receptor agonist with sedative, analgesic, anti-inflammatory and anti-sympathetic effects [5]. It has been widely used in clinical practice but its effect on diabetic nephropathy is currently unclear. In this study, a high-fat, high-sugar diet combined with low-dose streptozotocin (STZ) was used to establish the rat model of diabetes mellitus (DM). However, the effects and mechanisms of Dex in a DN treatment remained unclear until now.

## 2 Materials and Methods

### 2.1 Animals

60 SPF SD rats, 8-10 weeks old, 200~250g in weight, were raised in the Experimental Animal Center of Xinjiang Medical University, in an environment of 20±2 °C, 45±5% relative humidity, 12h/12h day/night shift, and 24/7 ventilation. The rats were caged in groups of 5, with free access to food and water. Rats of the normal control group were fed with basic diet, and rats in the high-fat group were fed with high-fat, high-sugar diets containing 20% sucrose, 10% lard, 2.5% cholesterol, 0.5% sodium cholate, and 67% basic diet.

### 2.2 Main reagents and instruments

STZ (Sigma, USA), Dex (Jiangsu Hengrui Pharmaceutical Co., Ltd.), Accu. Chek blood glucose meter (Roch, Germany), blood glucose test strip (Roch, Germany), radioimmunoassay kit for urine microalbumin, α1-microglobulin (α1-MG), β2-microglobulin (β2-MG) (Northern Biotechnology Research Institute, Beijing), rabbit RhoA, P-MYPT, Nox4 (Wuhan Sanying). R-210 Rotary Evaporator (Essen, Germany), VTl200 Paraffin Slicer (Leica, Germany), 5424SZX7.1093 Optical Microscope (Olympus, Japan), Freeze Dryer (Shanghai Xiyu), Free Radon Detector (Hidex, Finland).

### 2.3 Grouping, modeling and drug administration

Rats were fed for 1 week before experiments for adaption and randomly divided into 6 groups according to their body weight: one as normal group (9 rats), and the other 5 groups (45 rats) as high-fat groups. The normal group was fed with a normal diet, and the high-fat groups were fed with a high-fat diet. Eight weeks later, rats of the highfat group were injected with STZ at 35 mg/kg intraperitoneally, and another 7 days later, blood sample was taken from the tail vein to test random blood glucose, and a level ≥16.7 mmol/L was considered successful establishment of the model. Five rats died during the modeling process. The model rats were divided into a model group, a positive-drug group, and high-, medium- and low-Dex groups. Rats of the positive-drug group were administered with metformin at 200 mg/kg by gavage, the high-, medium- and low-Dex groups were given Dex at 1, 5 and 10μg/kg, and the normal control and model groups were given equal volume of normal saline. The dosing volume was 250 mL/kg, and the rats were treated for 4 weeks. All rats were fasted for 12 h before anatomy and sampling, then the rats were anesthetized with 10% chloral hydrate, blood was taken from heart and the kidneys were sampled. The blood was placed at room temperature for 2 h, then centrifuged at 4 °C, 3500 r/min for 15 min, and the upper serum was collected and stored at -20 °C for spare. The kidneys were weighed, fixed with 4% neutral paraformaldehyde, embedded in paraffin, and made into 5 μm paraffin sections.

This study was approval by Ethics committee of Fenghua District People's Hospital.

### 2.4 HE staining

The sections were dewaxed with xylene, stained according to the instructions of the HE kit, and observed under a biological microscope to access renal damage.

### 2.5 Masson staining

After 4 weeks of intervention, the kidneys of the rats were paraffin-embedded, sliced and deparaffinized to water, stained with hematoxylin for 5 min, washed twice in 95% ethanol, and incubated in picric acid/ethanol solution for 1 min for color separation. The sections were then rinsed with tap water and distilled water for 10 min, stained with acidic Ponceau solution, then with phosphomolybdic acid for 5 min and aniline acetate solution for 10 min, and at last dehydrated in gradient ethanol solutions, cleared, air dried, and mounted. Collagen fibers were stained blue, the muscle fibers, cellulose and red blood cells appeared red, and the nucleus looked dark blue. Thirty glomeruli were randomly selected from each section, and the stained area of collagen fibers in each glomerulus was scored as follows: 0 points for <25% stained area of glomerular collagen fiber; 1 point for 25%~50% stained area of glomerular collagen fiber; 2 points for 50%~75% stained area of glomerular collagen fiber; and 3 points for 75% ~100% stained area of glomerular collagen fiber.

### 2.6 Immunohistochemistry (IHC)

The sections were dewaxed, hydrated and washed with water, then blocked with sheep serum for 30 min. Antibodies for RhoA, P.MYPT, NOX4, α-SMA were diluted with TBST at 1:200, and a drop of the antibody solution was added to cover the sections and incubated in a humidity chamber at 4 °C overnight. After standing at room temperature for 1h, the slides were washed, and drops of secondary antibody were added and incubated at 37 °C for 30 min. The sections were then developed using the chromogenic reagents, counterstained with hematoxylin, blued with PBS, and sealed with natural resin. After staining, the nucleus appeared blue and the positively-stained area was brownish-yellow. The stained sections were then observed and photographed under a microscope. Image J software was used for analysis of all images.

### 2.7 Statistical analysis

SPSS l6.0 statistical software was used for data analysis. The experimental data were represented as Mean ± SD, and comparison between two groups was performed using independent-sample t test. P < 0.05 was considered statistically significant.

## 3 Results

### 3.1 Effect of Dex on the markers of renal damage in the model rats

Compared with the normal group, urine protein level of the model group significantly increased (P<0.01). Compared with the model group, the urine microalbumin level and early renal damage index α1-MG and β2-MG in the medium- and high-dose Dex groups significantly decreased (P<0.05 and P<0.01) as reported in Table 1.

**Table 1:** Levels of markers of renal injury in rats of each group (Mean±SD, n=9)

Group	Dose (mg/kg)	n	Urinary microalbumin(mg/24h)	α1-MG(ng/L)	β2-MG(ng/L)
NC		9	5.07±0.51	20.49±1.38	54.43±3.51
Model		9	34.38±1.18##	631.71±16.04##	94.30±2.65##
Dex-L	1μg/kg	9	29.85±0.88*	585.18±39.46**	85.66±4.82*
Dex-M	5μg/kg	9	28.85±1.38*	571.15±41.64**	83.24±4.48*
Dex-H	10μg/kg	9	26.31±1.97**	546.84±44.29**	79.32±6.21**
Met	200mg/kg	9	26.63±2.08**	532.06±52.13**	78.86±5.44**

##: P＜0.01, compared with NC group; *: P＜0.05, **: P＜0.01，***：P＜0.001, compared with Model group

### 3.2 Dex attenuates renal damage in the rat model of diabetic nephropathy

**Figure 1:**
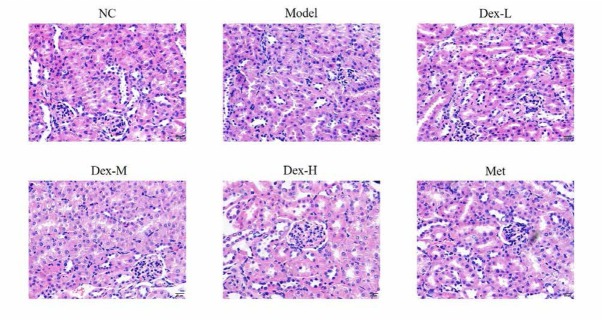
The Pathology of different groups in kidney tissues by HE staining (400×) NC: normal control group; Model: DN model group; Dex-L: Dex low-dose treated group; Dex-M: Dex medium-dose treated group; Dex-H: Dex high-dose treated group; Met: Metformin treated group

As shown in Figure 1, the control group showed normal renal tubular and glomerular tissues, while the model group showed obvious hyperplasia in the glomerular mesangial, thickened glomerular basement membrane, shrinkage of the glomerular capsule, capillary blockage, and significant renal fibrosis, indicating that the model was successfully established. Dex treatment alleviated renal damage, with the most significant effect in the 10μg/ kg group.

### 3.3 Effect of Dex on collagen deposition in rat kidney

**Figure 2:**
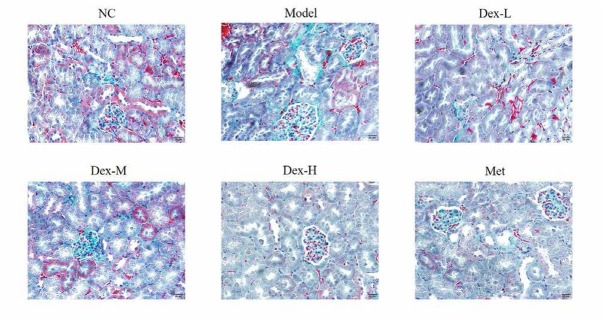
The Collagen fiber of different groups in kidney tissues by Masson staining (400×). NC: normal control group; Model: DN model group; Dex-L: Dex low-dose treated group; Dex-M: Dex medium-dose treated group; Dex-H: Dex high-dose treated group; Met: Metformin treated group

**Figure 3:**
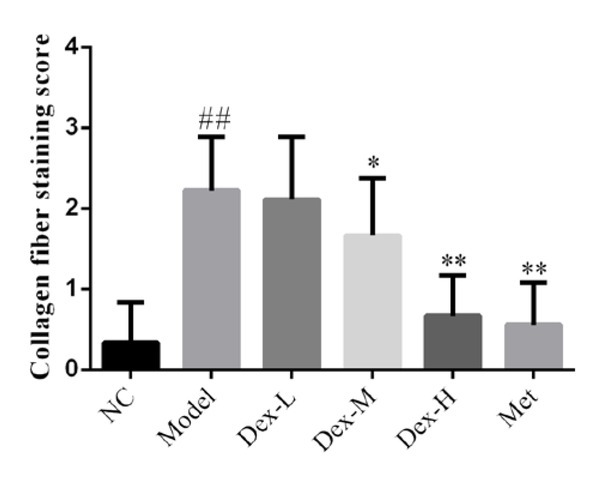
Comparison of Collagen Fiber Staining Rate in Kidney of Rats in Each Group NC: normal control group; Model: DN model group; Dex-L: Dex low-dose treated group; Dex-M: Dex medium-dose treated group; Dex-H: Dex high-dose treated group; Met: Metformin treated group ##: P<0.01, compared with NC group; *: P<0.05, **: P<0.01, compared with Model group

Masson staining demonstrated that collagen deposition in glomerular, renal tubule and interstitial of the control rats were basically normal; compared to the control group, rats of the model group showed significantly thickened glomerular basement membrane, increased collagen deposition in renal interstitial, and thickening of some renal tubule basement membrane with certain degree of dilatation and vacuolar-like lesions (P<0.01 for all); and compared to the model group, rats of the medium- and high-Dex groups showed significant improvements in collagen deposition in glomerular and interstitial areas (P<0.05 and P<0.01, respectively) (Figure 2 and Figure 3).

### 3.4 Effects of Dex on the expression of RhoA, p-MYPT and Nox4 proteins in rat kidney

**Figure 4:**
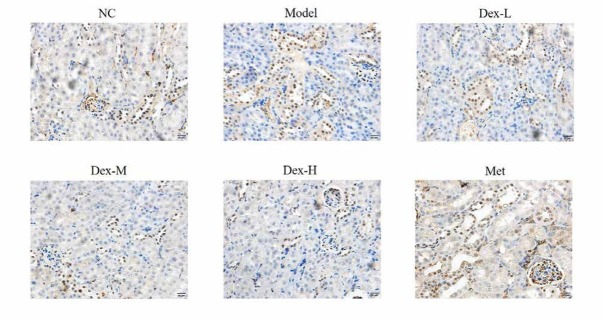
The RhoA protein expression of different groups by IHC assay (400×) NC: normal control group; Model: DN model group; Dex-L: Dex low-dose treated group; Dex-M: Dex medium-dose treated group; Dex-H: Dex high-dose treated group; Met: Metformin treated group

**Figure 5:**
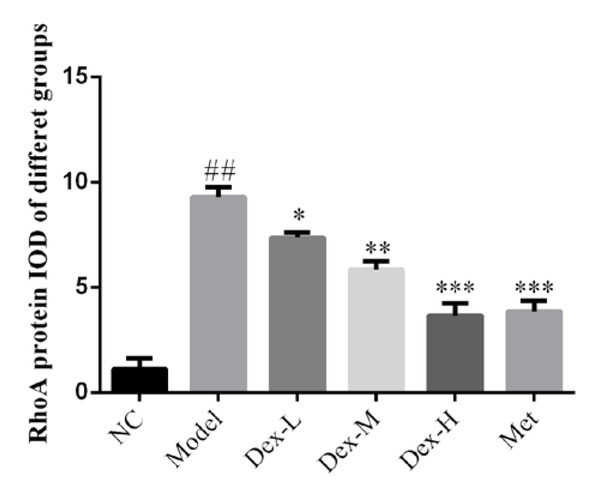
Comparison of RhoA protein expression in Kidney of Rats in Each Group NC: normal control group; Model: DN model group; Dex-L: Dex low-dose treated group; Dex-M: Dex medium-dose treated group; Dex-H: Dex high-dose treated group; Met: Metformin treated group ##: P<0.01, compared with NC group; *: P<0.05, **: P<0.01 ***: P<0.001, compared with Model group

**Figure 6:**
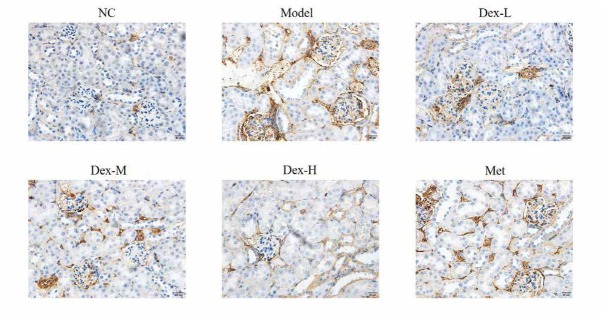
The p-MYPT protein expression of different groups by IHC assay (400×) NC: normal control group; Model: DN model group; Dex-L: Dex low-dose treated group; Dex-M: Dex medium-dose treated group; Dex-H: Dex high-dose treated group; Met: Metformin treated group

**Figure 7:**
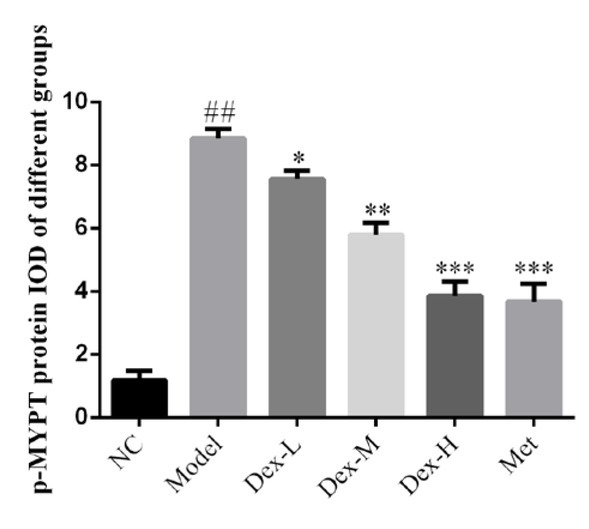
Comparison of p-MYPT protein expression in Kidney of Rats in Each Group NC: normal control group; Model: DN model group; Dex-L: Dex low-dose treated group; Dex-M: Dex medium-dose treated group; Dex-H: Dex high-dose treated group; Met: Metformin treated group ##: P<0.01, compared with NC group; *: P<0.05, **: P<0.01***, P<0.001, compared with Model group

**Figure 8:**
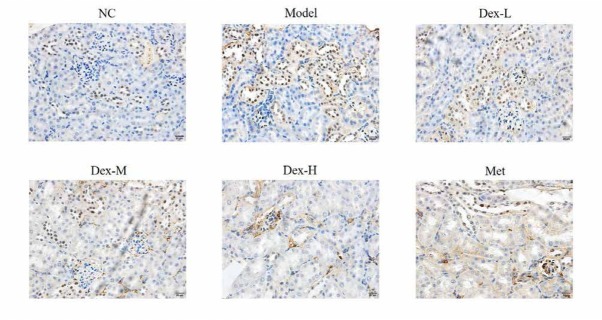
The Nox4 protein expression of different groups by IHC assay (400×) NC: normal control group; Model: DN model group; Dex-L: Dex low-dose treated group; Dex-M: Dex medium-dose treated group; Dex-H: Dex high-dose treated group; Met: Metformin treated

**Figure 9:**
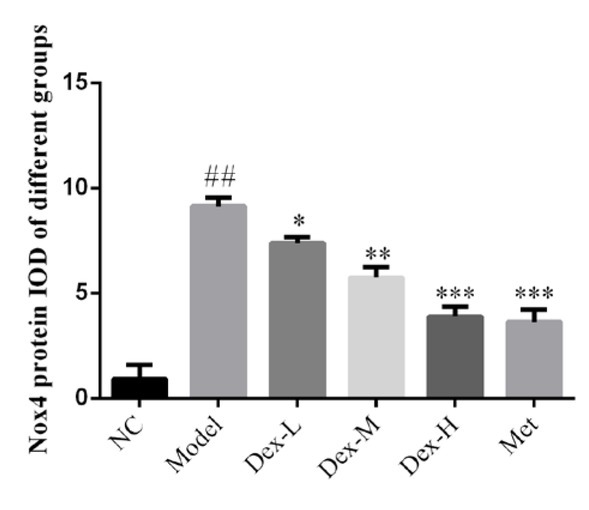
Comparison of p-MYPT protein expression in Kidney of Rats in Each Group NC: normal control group; Model: DN model group; Dex-L: Dex low-dose treated group; Dex-M: Dex medium-dose treated group; Dex-H: Dex high-dose treated group; Met: Metformin treated group ##: P<0.01, compared with NC group; *: P<0.05, **: P<0.01,*******: P<0.001, compared with Model group

Immunohistochemical staining showed that RhoA protein was mainly expressed in renal tubules of rat kidney, but no obvious expression was observed in glomerular region; p-MYPT protein was expressed in both renal tubules and glomeruli; Nox4 expression was observed in renal tubules and renal interstitial, and at significantly lower levels, in glomeruli. Expression of RhoA, p-MYPT and Nox4 protein in the model group significantly increased compared with that in the normal group, (P<0.01); and compared with the model group, expression of RhoA protein in the kidney tissue of the medium- and high-Dex groups significantly decreased (P<0.05); p-MYPT expression in the kidney tissue of the low-Dex group significantly decreased (P<0.01); and Nox4 expression significantly decreased in the low- and high-Dex groups (P<0.05 and P<0.01, respectively), (Figures 4-9).

### 3.5 Effect of Dex on protein expression of fibrin α-SMA in rat kidney tissues

**Figure 10:**
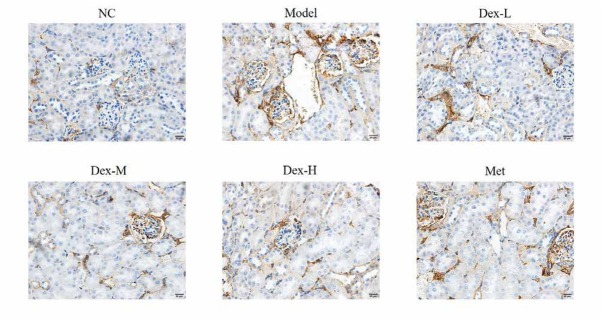
The α-SMA protein expression of different groups by IHC assay (400×) NC: normal control group; Model: DN model group; Dex-L: Dex low-dose treated group; Dex-M: Dex medium-dose treated group; Dex-H: Dex high-dose treated group; Met: Metformin treated

**Figure 11:**
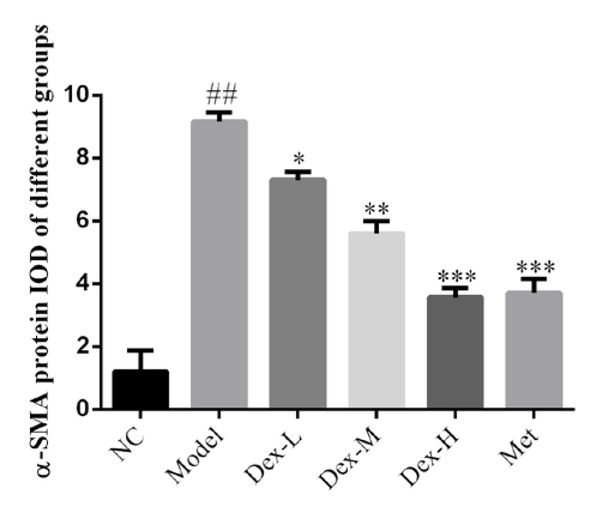
Comparison of α-SMA protein expression in Kidney of Rats in Each Group NC: normal control group; Model: DN model group; Dex-L: Dex low-dose treated group; Dex-M: Dex medium-dose treated group; Dex-H: Dex high-dose treated group; Met: Metformin treated group ##: P<0.01, compared with NC group; *: P<0.05, **: P<0.01***, P<0.001, compared with Model group

Immunohistochemical staining showed that fibrin α-SMA was mainly expressed in glomeruli and most prominently renal tubules of rat kidney. Compared with the normal group, α-SMA expression of the model group significantly increased (P<0.01); and compared with the model group, kidney α-SMA expression of the middle-Dex group significantly decreased (P<0.01), (Figure 10 and Figure 11).

## 4 Discussion

Diabetic nephropathy (DN) is a severe and most common microvascular complication of diabetes. It is a secondary renal disease characterized by renal fibrosis and represents a major cause of end-stage renal disease [5]. The pathogenesis of DN is complex and has not yet been fully elucidated. But it is currently believed that the pathogenesis of diabetes involves disorders of glucose and lipid metabolism, insulin resistance, changes in blood rheology, aggregation of glycosylation end products, and consequently oxidative stress in the kidney, which are closely related to the occurrence and development of DN [6, 7, 8, 9].

Urinary microalbumin is a major serum protein. Under normal circumstances, microalbumin can pass through glomerular basement membrane, then be reabsorbed by the proximal convoluted tubule, and elevated urinary microalbumin represents an important marker of early tubular damage caused by DM. In this study, low-, medium- and high-dose Dex all significantly inhibited 24-hour urinary albumin excretion in the DM rats, suggesting alleviation of early tubular injury. However, microalbumin is not a specific indicator of renal damage, as urinary tract inflammation, hemorrhage, fever, and stress can also increase the level of microalbumin. Therefore, other biomarkers are needed to further confirm the renal damage [10]. β2-MG is a polypeptide protein that exists ubiquitously, and α1-MG is a glycoprotein. Both proteins can be filtered through the glomerulus but are mostly reabsorbed and dissimilated in the renal tubules, so they exist at very low levels in urine of healthy human. In the early stages of renal damage, due to glomerular hyperfiltration and decreased reabsorption and dissimilation by the renal tubular, both β2-MG and α1-MG significantly increase in urine, representing a highly sensitive and specific marker of early renal damage [11, 12]. Since our model mainly simulates the pathophysiological characteristics of early DN, it would be rational to choose β2-MG and α1-MG as the indexes of successful model construction. Our results showed that high-, medium- and low-dose Dex significantly inhibited β2-MG and α1-MG levels in rat urine, indicating that Dex prevented and delayed early renal dysfunction in DM rats. Masson staining highlights deposition of immune complexes on the glomerulus, basement membrane, mesangial matrix, and collagen fibers, and is crucial for assessment of renal fibrotic lesions [13]. Our results showed that Dex significantly inhibited deposition of fibrous material in rat kidney tissue.

The molecular mechanism of DN oxidative stress is complex and has not yet been fully elucidated. In recent years, the effect of RhoA/ROCK signaling pathway on DN has received extensive attention [14]. RhoA is a member of the Rho-GTPase family, it is known as the “molecular regulatory hub” that plays a role in the regulation of multiple biological activities by binding to GTP or GDP. ROCK is a serine/threonine kinase right downstream of RhoA, and its activity can be reflected by level of p-MYPT. In kidney of STZ-induced diabetic rats and high glucose-treated mesangial cells, expression of RhoA on the cell membrane is significantly upregulated, and the RhoA/ROCK signaling pathway can also be activated by oxidative stress and hyperglycemia. Nox4 is the main subunit of NADPH oxidase, and is widely expressed in mesangial cells, podocytes, and renal tubular cells of rat and mouse models of DN. It has been reported that activation of RhoA/ROCK signaling pathway can induce the release of a large number of reactive oxygen species in various cells of kidney tissue via activation of Nox4, leading to expression of various fibrotic proteins such as α-SMA and type IV collagen [15, 16]. Immunohistochemical staining showed that Dex may inhibit expression of α-SMA in various cells such as mesangial cells, podocytes and endothelial cells through the RhoA/ROCK/Nox4 signaling pathway, thus delay fibrin deposition in renal tissue and prevent kidney fibrosis in DM rats. However, due to the limited spatial resolution of immunohistochemistry to detect protein expression in renal tissue, the specific site of action and target of Dex in rat kidney tissue, have not been revealed. Later, we shall further study the effect of Dex on expression of fibrotic protein in different cells of rat kidney tissue to further clarify its mechanism of action.
